# Dimorpholinium penta­chloridoanti­monate(III)

**DOI:** 10.1107/S1600536809019345

**Published:** 2009-05-29

**Authors:** Li Zhuang Chen

**Affiliations:** a Ordered Matter Science Research Center, College of Chemistry and Chemical Engineering, Southeast University, Nanjing 210096, People’s Republic of China

## Abstract

The asymmetric unit of the title compound, (C_4_H_10_NO)_2_[SbCl_5_], consists of two morpholinium cations in chair conformations, and a penta­chloridoanti­monate dianion with the Sb^III^ ion in a slightly distorted square-pyramidal coordination environment. The morpholinium cations are connected to each other by N—H⋯O hydrogen bonds, and they link the chloride anions and the anti­monate SbCl_3_ group *via* N—H⋯Cl contacts.

## Related literature

For a phase transition in bis­(ethyl­dimethyl­ammonium) penta­chlorido­anti­monate(III), see: Bujak & Zaleski (1999 ▶); for the structure of *N*-methyl­piperazinediium penta­chlorido­­anti­monate(III), see: Shen-Tu *et al.* (2008 ▶); for the low-temperature phase of morpholinium tetra­fluorido­borate, see: Owczarek *et al.* (2008 ▶).
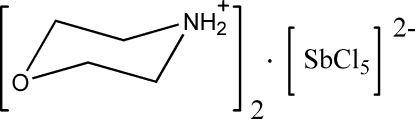


## Experimental

### 

#### Crystal data

(C_4_H_10_NO)_2_[SbCl_5_]
*M* *_r_* = 475.26
Orthorhombic, 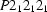

*a* = 9.0562 (18) Å
*b* = 10.273 (2) Å
*c* = 18.032 (4) Å
*V* = 1677.6 (6) Å^3^
*Z* = 4
Mo *K*α radiation
μ = 2.44 mm^−1^
*T* = 298 K
0.25 × 0.20 × 0.20 mm

#### Data collection

Rigaku Mercury2 (2× 2 bin mode) diffractometer
Absorption correction: multi-scan (*CrystalClear*; Rigaku, 2005 ▶) *T*_min_ = 0.567, *T*_max_ = 0.616
17552 measured reflections
3845 independent reflections
3759 reflections with *I* > 2σ(*I*)
*R*_int_ = 0.026

#### Refinement

*R*[*F* ^2^ > 2σ(*F* ^2^)] = 0.021
*wR*(*F* ^2^) = 0.046
*S* = 1.24
3845 reflections
163 parameters
H-atom parameters constrained
Δρ_max_ = 0.32 e Å^−3^
Δρ_min_ = −0.66 e Å^−3^
Absolute structure: Flack (1983 ▶)
Flack parameter: −0.005 (15)

### 

Data collection: *CrystalClear* (Rigaku, 2005 ▶); cell refinement: *CrystalClear*; data reduction: *CrystalClear*; program(s) used to solve structure: *SHELXS97* (Sheldrick, 2008 ▶); program(s) used to refine structure: *SHELXL97* (Sheldrick, 2008 ▶); molecular graphics: *SHELXTL* (Sheldrick, 2008 ▶); software used to prepare material for publication: *SHELXL97*.

## Supplementary Material

Crystal structure: contains datablocks I, global. DOI: 10.1107/S1600536809019345/si2174sup1.cif

Structure factors: contains datablocks I. DOI: 10.1107/S1600536809019345/si2174Isup2.hkl

Additional supplementary materials:  crystallographic information; 3D view; checkCIF report

## Figures and Tables

**Table 1:** Hydrogen-bond geometry (Å, °)

*D*—H⋯*A*	*D*—H	H⋯*A*	*D*⋯*A*	*D*—H⋯*A*
N1—H1*D*⋯Cl5^i^	0.90	2.35	3.180 (2)	154
C1—H1*B*⋯Cl3^ii^	0.97	2.82	3.548 (3)	132
N2—H2*D*⋯Cl5^iii^	0.90	2.73	3.394 (2)	131
N2—H2*C*⋯Cl1^iv^	0.90	2.45	3.306 (3)	159
N2—H2*D*⋯O1^v^	0.90	2.44	2.848 (3)	108
N1—H1*C*⋯Cl3	0.90	2.75	3.463 (3)	137
N1—H1*C*⋯Cl5	0.90	2.72	3.448 (3)	138

Symmetry codes: (i) 
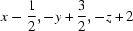
; (ii) 
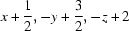
; (iii) 
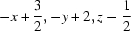
; (iv) 
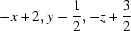
; (v) 
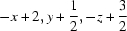
.

**Fig. 1.**
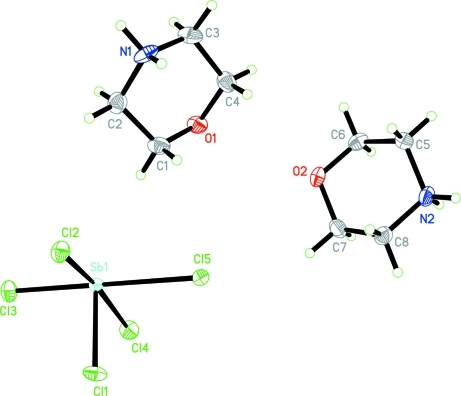
A view of the title compound with the atomic numbering scheme. Displacement ellipsoids were drawn at the 30% probability level.

**Fig. 2.**
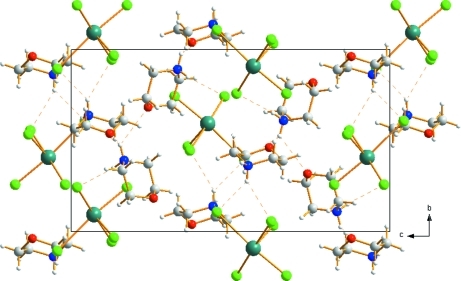
The packing viewed approximately along the b axis. Hydrogen bonds are drawn as dashed lines.

## supplementary crystallographic information

### Comment

Structural investigation of crystalline solids undergoing phase transformation has been one of the classical areas of research among both chemists and physicists. The morpholinium tetrafluoroborate undergoes two reversible phase transitions (Owczarek *et al.* 2008). In our laboratory, a compound containing two morpholinium cations and a pentachloridoantimonate dianion in the asymmetric unit has been synthesized (Fig. 1), with the Sb^III^ ion in a slightly distorted square-pyramidal coordination environment.

The Sb atom is coordinated by five Cl atoms, with Sb—Cl distances ranging from 2.4045 (8) to 2.9230 (9) Å. The Sb—Cl distances are similar to the values of 2.4110 (10) to 2.9112 (11) Å reported by Shen-Tu *et al.* (2008) and slightly different to the values of 2.499 (4)–2.768 (4) Å reported by Bujak & Zaleski (1999). In the title compound the difference between the longest bond (Sb1—Cl5) and shortest bond (Sb1—Cl4) is *ca* 0.50 Å. The six-membered ring morpholinium cations have chair conformation. The morpholinium cations are connected to each other by N—H···O hydrogen bonds, and they link the Cl^-^ anions and the antimonate group SbCl_3_*via* N–H···Cl contacts (Table 1, Fig. 2).

### Experimental

SbCl_3_, morpholine and 20% aqueous HCl in a molar ratio of 1:1:1 were mixed and dissolved in sufficient ethanol by heating to 353 K forming a clear solution. The reaction mixture was cooled slowly to room temperature, crystals of the title compound were formed, collected and washed with dilute aqueous HCl.

### Refinement

H atoms were included in calculated positions with N—H = 0.90 and C—H = 0.97 Å and included in the riding-model approximation with *U*_iso_(H) = 1.2*U*_eq_(C, N).

### Crystal data

**Table:**

(C_4_H_10_NO)_2_[SbCl_5_]	*F*(000) = 936
*M**_r_* = 475.26	*D*_x_ = 1.882 Mg m^−^^3^
Orthorhombic, *P*2_1_2_1_2_1_	Mo *K*α radiation, λ = 0.71073 Å
Hall symbol: P 2ac 2ab	Cell parameters from 3845 reflections
*a* = 9.0562 (18) Å	θ = 3–27.5°
*b* = 10.273 (2) Å	µ = 2.44 mm^−^^1^
*c* = 18.032 (4) Å	*T* = 298 K
*V* = 1677.6 (6) Å^3^	Block, colourless
*Z* = 4	0.25 × 0.20 × 0.20 mm

### Data collection

**Table:**

Rigaku Mercury2 (2× 2 bin mode) diffractometer	3845 independent reflections
Radiation source: fine-focus sealed tube	3759 reflections with *I* > 2σ(*I*)
graphite	*R*_int_ = 0.026
Detector resolution: 13.6612 pixels mm^-1^	θ_max_ = 27.5°, θ_min_ = 3.0°
ω scans	*h* = −11→11
Absorption correction: multi-scan (*CrystalClear*; Rigaku, 2005)	*k* = −13→13
*T*_min_ = 0.567, *T*_max_ = 0.616	*l* = −23→23
17552 measured reflections	

### Refinement

**Table:**

Refinement on *F*^2^	Secondary atom site location: difference Fourier map
Least-squares matrix: full	Hydrogen site location: inferred from neighbouring sites
*R*[*F*^2^ > 2σ(*F*^2^)] = 0.021	H-atom parameters constrained
*wR*(*F*^2^) = 0.046	*w* = 1/[σ^2^(*F*_o_^2^) + (0.0201*P*)^2^] where *P* = (*F*_o_^2^ + 2*F*_c_^2^)/3
*S* = 1.24	(Δ/σ)_max_ = 0.003
3845 reflections	Δρ_max_ = 0.32 e Å^−^^3^
163 parameters	Δρ_min_ = −0.66 e Å^−^^3^
0 restraints	Absolute structure: Flack (1983)
Primary atom site location: structure-invariant direct methods	Flack parameter: −0.005 (15)

### Special details

**Table:**

Geometry. All esds (except the esd in the dihedral angle between two l.s. planes) are estimated using the full covariance matrix. The cell esds are taken into account individually in the estimation of esds in distances, angles and torsion angles; correlations between esds in cell parameters are only used when they are defined by crystal symmetry. An approximate (isotropic) treatment of cell esds is used for estimating esds involving l.s. planes.
Refinement. Refinement of F^2^ against ALL reflections. The weighted R-factor wR and goodness of fit S are based on F^2^, conventional R-factors R are based on F, with F set to zero for negative F^2^. The threshold expression of F^2^ > 2sigma(F^2^) is used only for calculating R-factors(gt) etc. and is not relevant to the choice of reflections for refinement. R-factors based on F^2^ are statistically about twice as large as those based on F, and R- factors based on ALL data will be even larger.

### Fractional atomic coordinates and isotropic or equivalent isotropic displacement parameters (Å^2^)

**Table:**

	*x*	*y*	*z*	*U*_iso_*/*U*_eq_	
C1	0.7135 (3)	0.5818 (4)	0.95313 (17)	0.0425 (8)	
H1A	0.7413	0.6730	0.9546	0.051*	
H1B	0.7866	0.5329	0.9808	0.051*	
C2	0.5653 (4)	0.5651 (3)	0.98887 (17)	0.0431 (7)	
H2A	0.5395	0.4735	0.9906	0.052*	
H2B	0.5680	0.5980	1.0393	0.052*	
C3	0.4560 (3)	0.5973 (4)	0.86593 (18)	0.0459 (8)	
H3A	0.3890	0.6514	0.8374	0.055*	
H3B	0.4236	0.5076	0.8616	0.055*	
C4	0.6092 (3)	0.6104 (3)	0.83609 (17)	0.0440 (8)	
H4A	0.6114	0.5797	0.7852	0.053*	
H4B	0.6372	0.7016	0.8361	0.053*	
C5	0.8890 (4)	0.6967 (3)	0.68509 (19)	0.0461 (8)	
H5A	0.7903	0.6605	0.6819	0.055*	
H5B	0.9521	0.6478	0.6516	0.055*	
C6	0.8854 (3)	0.8366 (3)	0.66122 (19)	0.0442 (8)	
H6A	0.8495	0.8431	0.6107	0.053*	
H6B	0.8196	0.8858	0.6931	0.053*	
C7	1.0960 (3)	0.8730 (3)	0.74228 (15)	0.0376 (6)	
H7A	1.0382	0.9243	0.7769	0.045*	
H7B	1.1976	0.9028	0.7446	0.045*	
C8	1.0880 (4)	0.7312 (3)	0.76295 (16)	0.0411 (7)	
H8A	1.1510	0.6814	0.7299	0.049*	
H8B	1.1247	0.7199	0.8131	0.049*	
Cl1	0.82449 (9)	1.24256 (9)	0.98134 (4)	0.0472 (2)	
Cl2	0.55039 (9)	1.24280 (7)	0.82480 (4)	0.04292 (19)	
Cl3	0.39146 (8)	0.94495 (7)	0.87224 (4)	0.03967 (18)	
Cl4	0.76734 (9)	0.96688 (8)	0.86621 (5)	0.04330 (19)	
Cl5	0.60479 (7)	0.89292 (7)	1.04341 (4)	0.03298 (15)	
N1	0.4534 (2)	0.6378 (2)	0.94513 (14)	0.0407 (6)	
H1C	0.4718	0.7238	0.9483	0.049*	
H1D	0.3631	0.6231	0.9641	0.049*	
N2	1.0376 (3)	0.8903 (2)	0.66613 (14)	0.0365 (6)	
H2C	1.0966	0.8490	0.6336	0.044*	
H2D	1.0366	0.9754	0.6544	0.044*	
O1	0.7123 (2)	0.5387 (2)	0.87871 (12)	0.0377 (5)	
O2	0.9422 (2)	0.6830 (2)	0.75855 (11)	0.0430 (5)	
Sb1	0.580748 (19)	1.092473 (15)	0.928491 (9)	0.02418 (5)	

### Atomic displacement parameters (Å^2^)

**Table:**

	*U*^11^	*U*^22^	*U*^33^	*U*^12^	*U*^13^	*U*^23^
C1	0.0334 (15)	0.054 (2)	0.0404 (17)	0.0040 (17)	−0.0037 (13)	−0.0141 (16)
C2	0.0470 (18)	0.0450 (18)	0.0374 (16)	−0.0036 (16)	0.0051 (15)	−0.0036 (13)
C3	0.0338 (16)	0.053 (2)	0.0507 (19)	0.0112 (17)	−0.0098 (13)	−0.0112 (17)
C4	0.0459 (18)	0.0497 (19)	0.0364 (16)	0.0138 (17)	0.0012 (13)	0.0029 (14)
C5	0.0397 (18)	0.0457 (18)	0.0531 (19)	−0.0173 (15)	−0.0041 (15)	0.0022 (15)
C6	0.0322 (16)	0.0501 (19)	0.0502 (19)	−0.0012 (15)	−0.0070 (14)	0.0157 (15)
C7	0.0376 (15)	0.0393 (15)	0.0360 (15)	−0.0078 (14)	0.0006 (13)	−0.0015 (12)
C8	0.0445 (17)	0.0466 (17)	0.0322 (15)	0.0030 (17)	−0.0056 (15)	0.0060 (12)
Cl1	0.0464 (4)	0.0481 (5)	0.0472 (5)	−0.0051 (4)	0.0000 (4)	−0.0024 (4)
Cl2	0.0547 (5)	0.0364 (4)	0.0377 (4)	−0.0036 (4)	−0.0052 (3)	0.0098 (3)
Cl3	0.0387 (4)	0.0398 (4)	0.0405 (4)	−0.0074 (3)	−0.0098 (3)	0.0001 (3)
Cl4	0.0394 (4)	0.0377 (4)	0.0529 (5)	0.0030 (4)	0.0191 (4)	−0.0080 (3)
Cl5	0.0284 (3)	0.0367 (4)	0.0338 (3)	0.0025 (3)	0.0000 (3)	0.0024 (3)
N1	0.0215 (11)	0.0392 (14)	0.0613 (18)	−0.0020 (10)	0.0108 (11)	−0.0130 (12)
N2	0.0381 (13)	0.0300 (13)	0.0413 (14)	−0.0037 (11)	0.0054 (10)	0.0085 (11)
O1	0.0298 (11)	0.0443 (13)	0.0389 (12)	0.0123 (9)	0.0013 (9)	−0.0074 (10)
O2	0.0443 (12)	0.0420 (11)	0.0427 (12)	−0.0127 (11)	0.0039 (10)	0.0146 (9)
Sb1	0.02303 (8)	0.02452 (8)	0.02498 (8)	−0.00036 (8)	0.00101 (7)	−0.00180 (7)

### Geometric parameters (Å, °)

**Table:**

C1—O1	1.413 (4)	C6—N2	1.487 (4)
C1—C2	1.499 (4)	C6—H6A	0.9700
C1—H1A	0.9700	C6—H6B	0.9700
C1—H1B	0.9700	C7—N2	1.482 (4)
C2—N1	1.485 (4)	C7—C8	1.506 (4)
C2—H2A	0.9700	C7—H7A	0.9700
C2—H2B	0.9700	C7—H7B	0.9700
C3—N1	1.488 (4)	C8—O2	1.412 (4)
C3—C4	1.494 (4)	C8—H8A	0.9700
C3—H3A	0.9700	C8—H8B	0.9700
C3—H3B	0.9700	Cl1—Sb1	2.8562 (9)
C4—O1	1.416 (4)	Cl2—Sb1	2.4405 (8)
C4—H4A	0.9700	Cl3—Sb1	2.5028 (8)
C4—H4B	0.9700	Cl4—Sb1	2.4045 (8)
C5—O2	1.417 (4)	N1—H1C	0.9000
C5—C6	1.501 (4)	N1—H1D	0.9000
C5—H5A	0.9700	N2—H2C	0.9000
C5—H5B	0.9700	N2—H2D	0.9000
			
O1—C1—C2	111.4 (2)	C5—C6—H6B	110.0
O1—C1—H1A	109.3	H6A—C6—H6B	108.4
C2—C1—H1A	109.3	N2—C7—C8	109.1 (2)
O1—C1—H1B	109.3	N2—C7—H7A	109.9
C2—C1—H1B	109.3	C8—C7—H7A	109.9
H1A—C1—H1B	108.0	N2—C7—H7B	109.9
N1—C2—C1	109.0 (3)	C8—C7—H7B	109.9
N1—C2—H2A	109.9	H7A—C7—H7B	108.3
C1—C2—H2A	109.9	O2—C8—C7	111.7 (3)
N1—C2—H2B	109.9	O2—C8—H8A	109.3
C1—C2—H2B	109.9	C7—C8—H8A	109.3
H2A—C2—H2B	108.3	O2—C8—H8B	109.3
N1—C3—C4	109.6 (2)	C7—C8—H8B	109.3
N1—C3—H3A	109.8	H8A—C8—H8B	107.9
C4—C3—H3A	109.8	C2—N1—C3	111.0 (2)
N1—C3—H3B	109.8	C2—N1—H1C	109.4
C4—C3—H3B	109.8	C3—N1—H1C	109.4
H3A—C3—H3B	108.2	C2—N1—H1D	109.4
O1—C4—C3	111.7 (3)	C3—N1—H1D	109.4
O1—C4—H4A	109.3	H1C—N1—H1D	108.0
C3—C4—H4A	109.3	C7—N2—C6	110.0 (2)
O1—C4—H4B	109.3	C7—N2—H2C	109.7
C3—C4—H4B	109.3	C6—N2—H2C	109.7
H4A—C4—H4B	107.9	C7—N2—H2D	109.7
O2—C5—C6	111.7 (3)	C6—N2—H2D	109.7
O2—C5—H5A	109.3	H2C—N2—H2D	108.2
C6—C5—H5A	109.3	C1—O1—C4	111.0 (2)
O2—C5—H5B	109.3	C8—O2—C5	109.6 (2)
C6—C5—H5B	109.3	Cl4—Sb1—Cl2	93.49 (3)
H5A—C5—H5B	107.9	Cl4—Sb1—Cl3	88.12 (3)
N2—C6—C5	108.5 (2)	Cl2—Sb1—Cl3	89.74 (3)
N2—C6—H6A	110.0	Cl4—Sb1—Cl1	84.40 (3)
C5—C6—H6A	110.0	Cl2—Sb1—Cl1	90.06 (3)
N2—C6—H6B	110.0	Cl3—Sb1—Cl1	172.49 (3)

### Hydrogen-bond geometry (Å, °)

**Table:**

*D*—H···*A*	*D*—H	H···*A*	*D*···*A*	*D*—H···*A*
N1—H1D···Cl5^i^	0.90	2.35	3.180 (2)	154
C1—H1B···Cl3^ii^	0.97	2.82	3.548 (3)	132
N2—H2D···Cl5^iii^	0.90	2.73	3.394 (2)	131
N2—H2C···Cl1^iv^	0.90	2.45	3.306 (3)	159
N2—H2D···O1^v^	0.90	2.44	2.848 (3)	108
N1—H1C···Cl3	0.90	2.75	3.463 (3)	137
N1—H1C···Cl5	0.90	2.72	3.448 (3)	138

Symmetry codes: (i) *x*−1/2, −*y*+3/2, −*z*+2; (ii) *x*+1/2, −*y*+3/2, −*z*+2; (iii) −*x*+3/2, −*y*+2, *z*−1/2; (iv) −*x*+2, *y*−1/2, −*z*+3/2; (v) −*x*+2, *y*+1/2, −*z*+3/2.
